# Bio-Inspired Neural Network for Real-Time Evasion of Multi-Robot Systems in Dynamic Environments

**DOI:** 10.3390/biomimetics9030176

**Published:** 2024-03-15

**Authors:** Junfei Li, Simon X. Yang

**Affiliations:** School of Engineering, University of Guelph, 50 Stone Road East, Guelph, ON N1G2W1, Canada; jli64@uoguelph.ca

**Keywords:** bio-inspired algorithms, pursuit–evasion games, neurodynamic models

## Abstract

In complex and dynamic environments, traditional pursuit–evasion studies may face challenges in offering effective solutions to sudden environmental changes. In this paper, a bio-inspired neural network (BINN) is proposed that approximates a pursuit–evasion game from a neurodynamic perspective instead of formulating the problem as a differential game. The BINN is topologically organized to represent the environment with only local connections. The dynamics of neural activity, characterized by the neurodynamic shunting model, enable the generation of real-time evasive trajectories with moving or sudden-change obstacles. Several simulation and experimental results indicate that the proposed approach is effective and efficient in complex and dynamic environments.

## 1. Introduction

The pursuit–evasion game is a classic issue in robotics, in which one or more “pursuers” attempt to capture one or more “evaders” in different environments [1,2,3,4]. The traditional pursuit–evasion algorithms used differential game formulation for the modeling and analysis of the pursuer and evader [5,6,7]. As the number of agents involved in the pursuit–evasion game increases, the complexity of the problem increases. Tian et al. [8] proposed a distributed cooperative pursuit strategy for an evader in an obstacle-cluttered environment. Cheng and Yuan [9] proposed a parameter-adaptive method to update the pursuit and evasion strategies when considering collision avoidance in the multiplayer. Sani et al. [10] considered a pursuit–evasion game based on the nonlinear model predictive control in static obstacle environments, where the pursuer and the evader are nonholonomic mobile robots. The pursuit–evasion problem has a wide range of applications, including navigation [11], surveillance [12,13], human–robot cooperation [14] and robotic foraging behaviors [15].

The problem of multiple evaders against a single pursuer requires the pursuer to capture multiple evaders in a finite amount of time. Therefore, the pursuer typically has some advantages over the evaders, such as faster speed, greater maneuverability, or more information about the environment [16]. The main challenge for the evaders is to increase the survival time. One approach that has been developed to guide multiple evaders is to formulate them as a group moving together and following a single leader [17]. Scott and Leonard [18] analyzed a dynamic model of multiple heterogeneous evaders against a faster pursuer and presented pursuit and evasion strategies considering global or local detection. However, their study only considered collision-free obstacle environment. The LoS (line-of-sight) guidance principle has been widely used in the pursuit–evasion game due to its simplicity, intuition, and low computational burden [19]. However, a singular escape direction may lead the evader to a local minimum constituted by obstacles in complex environments. Scott and Leonard [20] proposed a strategy for evaders not initially targeted to avoid capture and considered the limited sensing condition through a local risk reduction strategy. However, they only considered that multiple evaders work in collision-free obstacle environments. Some studies focused on the influence of the obstacle in the pursuit-evasion game. The traditional methods impose some limitations to the physical states for both pursuers and evaders to formulate the game in a bounded area or obstacle environment. Fisac and Sastry [21] proposed a two-player game where the obstacle is able to delay or avoid capture from the pursuer. Oyler [22] considered the effect of asymmetric obstacles, where the obstacle has different influences on each player. Bhadauria and Isler [23] considered a bounded environment with some static obstacles. When considering the effect of the obstacle, the visibility may not be full for the pursuer and evader. Wu et al. [24] proposed a flock control algorithm that can guide swarming aerial vehicles with obstacle avoidance in a dynamic and unknown 3D environment. Dong et al. [25] incorporated the artificial potential field method to design a collision-free evasion model. Oyler et al. [22] proposed dominance regions to generate the evasion path considering the presence of obstacles. However, these studies often rely on assumptions about the behavior of the pursuer. These assumptions may not always hold in practice, which makes it difficult to develop strategies for unpredictable pursuit behavior.

Recently, the neural network approach has become a hot research topic [26,27]. Many studies considered using neural networks to deal with the pursuit–evasion problem. Qi et al. [28] proposed a deep Q-network approach to guide the evader to escape from the pursuer based on a self-play mechanism. Qu et al. [29] proposed a deep reinforcement learning approach to generate pursuit and evasion trajectories for unmanned surface vehicles. The proposed approach considered multi-obstacle influences in the water surface environment. Guo et al. [30] proposed a neural network-based control method to ensure that the velocities of the pursuer and the evader converge to their desired values with unknown dynamics. However, pursuit–evasion games that use learning-based neural network approaches are not efficient and computationally expensive, especially in their initial learning.

This paper aims to provide a novel approach that approximates a general pursuit–evasion game from a neurodynamic perspective instead of formulating the problem as a differential game. In this paper, the neurodynamics-based approach aims to overcome the limitations of the traditional approach and improve the performance of evaders in dynamic and uncertain environments. A bio-inspired neural network (BINN) is applied to guide robots to evade a single faster pursuer in the presence of dynamic obstacles. Compared with existing research, the contributions of this paper are summarized as follows:

(1) The concept of a pursuit–evasion game with sudden environmental changes is proposed for the first time.
(2) A novel neurodynamic-based approach is proposed to approximate the pursuit–evasion game instead of formulating the problem as a differential game.
(3) A novel real-time evasion strategy is proposed based on the landscape of the neural activity without any learning procedures.

This paper is organized as follows. Section 2 offers the preliminaries and a description of the problem. Section 3 describes the proposed approaches to evasion. Section 4 shows the simulation and experimental results involving different scenarios. In Section 5, the results of this research are briefly summarized.

## 2. Problem Statement

For a group of *m* evader robots, their time-varying location in the workspace, *W*, can be uniquely determined by the spatial position, $p_{e}=\left(x_{e},y_{e}\right)$, e=1,…,m. The time-varying position of the faster pursuer can be denoted by $p_{u}=\left(x_{u},y_{u}\right)$. Suppose the pursuer has full knowledge of the environment. However, the pursuer has no knowledge about the evasion strategy of evaders and their evasion directions. It is the same for the evader, which has full knowledge of the environment but has no information on the direction of the move and the pursuit strategy of the pursuer. Because the moving directions and strategies of the pursuer and evader are unknown to each other, the trajectories of the evader and pursuer are not able to predict depending on their kinematics model. Thus, the kinematics model of the pursuer and evader is not considered in this paper. In the workspace, *W*, there is a sequence of *i* obstacles, and their position can be denoted by $p_{o}=\left(x_{o},y_{o}\right)$, o=1,…,i. The speed of moving obstacles can be denoted by $v_{o}$. The boundary of the workspace is assumed to be a sequence of static obstacles. If the evader reaches the environmental limit, the evader is considered a collision with obstacles.

The speed of the pursuer is denoted by $v_{u}$, whereas the speed of the evader is denoted by $v_{e}$. Suppose that both the evader and the faster pursuer move with constant speeds. In the workspace, *W*, assume that the pursuer captures the evader at position *f* in limit time *T*. The position of *f* can be denoted by $f=(x_{f},y_{f})$. Position *f* satisfies the following condition:

(1) $$\frac{\sqrt{{\left(x_{f}-x_{u}\right)}^{2}+{\left(y_{f}-y_{u}\right)}^{2}}}{\sqrt{{\left(x_{f}-x_{e}\right)}^{2}+{\left(y_{f}-y_{e}\right)}^{2}}}=\frac{v_{u}T}{v_{e}T}=\lambda_{e},$$

where $\lambda_{e}=v_{u}/v_{e}$ is the speed ratio of evader and the pursuer. The speed ratio plays an important role in the evasion task [31]. If speed ratio $\lambda_{e}$ is very large, it means the evader can be easily captured by the pursuer. In this paper, the pursuer is faster than the evaders. Thus, $\lambda_{e}>1$ in all considerations. The purpose of evasion is to increase the total survival time $t_{s}$ of evaders. Since the pursuer has full knowledge of the environment and the pursuer and the evader move with constant speed, the strategy of the pursuer is equivalent to a multi-target path planning problem [22]. The number of evaders is greater than that of the pursuers; at each instant of time, the pursuer captures evaders based on the following sequence:

(2) $$\Psi_{t}=\min\left\{d_{\mathrm{eu}},e=1,2,\ldots ,m\right\},$$

where $\Psi_{t}$ is the evader assigned for the pursuer to capture at the instant of time *t*; and $d_{eu}=\left|\left(x_{e}-x_{u},y_{e}-y_{u}\right)\right|$ is the Euclidean distance between the pursuer and each evader. The pursuer captures the nearest robot until all the robots are captured. If two evaders are equidistant from the pursuer, a random evader is chosen for one of them.

Therefore, the problem studied in this paper can be described as follows: for a group of *m* evaders and one faster-moving pursuer, given the initial positions of evaders $p_{e}\left(0\right)$ with e=1,…,m and $p_{u}\left(0\right)$ with $∥p_{u}\left(0\right)-p_{e}\left(0\right)∥>d_{c}$, the collision-free trajectories of robots are generated to increase survival time $t_{s}$ for the group of evaders. The evader is captured by a faster pursuer when the distance between the purser and the evader is smaller than capture distance $d_{c}>0$.

Since several movable and sudden change obstacles exist in the workspace, the evaders should not only escape the pursuer, but also guarantee the evasion trajectories are real-time collision-free to obstacles. Note that the term “real-time” means that the response of the robot trajectory generator is instant to the dynamic environmental changes.

## 3. Proposed Approach

In the proposed approach, the environment is represented one to one by a neural network with only local connections. The evasion strategy is analyzed and designed from a neurodynamic perspective. The real-time collision-free evasion trajectories are generated through dynamics of neural activity.

### 3.1. Environment Representation via Bio-Inspired Neural Network

**Figure 1:**
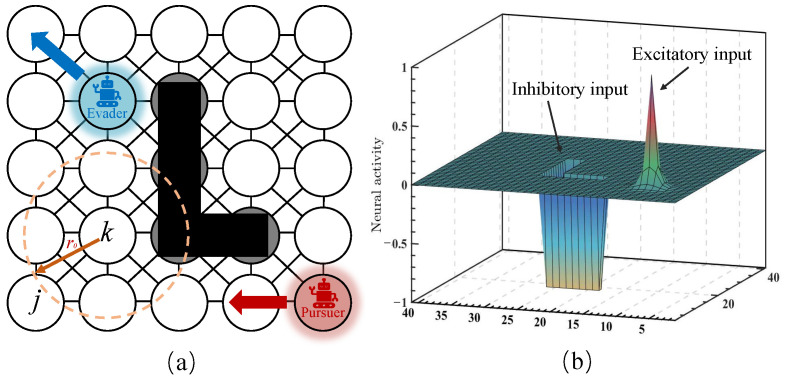
The example of the bio-inspired neural network. (**a**) Structure of the neural network with only local connections; (**b**) example of dynamic landscape of neural activity.

In this study, the fundamental idea is to construct a topologically organized neural network architecture in which the dynamic landscape of neural activity represents the dynamic environment. By properly defining the external inputs from the dynamic environment and internal neural connections, the pursuer and obstacles are guaranteed to stay at the peak and valley of the activity landscape of the neural network, respectively. The architecture of the proposed neural network is illustrated in Figure 1a. The proposed neural network is characterized by its local connectivity structure in which the neuron is only connected to other neurons within a small region $\left(0,r_{0}\right)$. The neighboring neurons are defined as those whose distance between the *k*th neuron and the *j*th neuron is smaller than $r_{0}$. The environment is assigned one to one to the neural network, as illustrated in Figure 1a. The black squares represent the position of the obstacles and the gray circles represent the corresponding neurons that map to the obstacles. The red square represents the position of the pursuer, and the blue square represents the position of the evader.

Hodgkin and Huxley [32] proposed an electrical circuit for modeling the membrane potential in a biological neuron system. Using the state equation technique, the dynamics of membrane $V_{m}$ can be obtained as

(3) $$\begin{array}{cc} C_{m}\frac{dV_{m}}{dt}= & -\left(E_{l}+V_{m}\right)g_{l}+\left(E_{Na}-V_{m}\right)g_{Na}-\left(E_{K}+V_{m}\right)g_{K}, \end{array}$$

where $C_{m}$ is the capacitance of the membrane; $E_{K}$ and $E_{Na}$ are the equilibrium potentials of potassium and sodium ions, respectively; $E_{l}$ is the potentials of passive leak current due to chloride and other ions; $g_{K}$ and $g_{Na}$ are the ionic conductances of the potassium and sodium, respectively; $g_{l}$ is conductances of chloride and other ions. Grossberg [33] developed a shunting neurodynamics model that establishes $C_{m}=1$ and substitutes $x_{k}=E_{p}+V_{m}$, $A=g_{l}$, $B=E_{Na}+E_{l}$, $D=E_{k}-E_{l}$, $S_{k}^{e}=g_{Na}$, and $S_{k}^{i}=g_{K}$ in (3). Therefore, the shunting equation can be written as

(4) $$\frac{dx_{k}}{dt}=-Ax_{k}+\left(B-x_{k}\right)S_{k}^{e}-\left(D+x_{k}\right)S_{k}^{i},$$

where $x_{k}$ denotes the neural activity of the *k*th neuron; $S_{k}^{e}$ and $S_{k}^{i}$ are the excitatory and inhibitory inputs to the neuron, respectively; *A* is the passive decay rate; *B* and *D* are the upper and lower bounds of the neural activity, respectively. The neural activity is bounded in the area of [−D,B]. Several robotic navigation and control algorithms have been developed depending on the neurodynamic shunting model [34,35,36].

Based on (4), the excitatory input $S_{k}^{e}$ is derived from the pursuer and its neighboring neurons, while the inhibitory input $S_{k}^{i}$ is derived from obstacles. Therefore, the neural activity for the *k*th neuron is written as

(5) $$\begin{array}{c} \frac{dx_{k}}{dt}=-Ax_{k}+\left(B-x_{k}\right)\left({\left[I_{k}\right]}^{+}+\sum_{j=1}^{n}w_{kj}{\left[x_{j}\right]}^{+}\right)-\left(D+x_{k}\right){\left[I_{k}\right]}^{-}, \end{array}$$

where $x_{j}$ represents the neural activity of neighboring neurons to the *k*th neuron; *n* represents the amount of neighboring neurons to the *k*th neuron; ${\left[a\right]}^{+}$ is defined as ${\left[a\right]}^{+}=\max\left\{0,a\right\}$; and ${\left[a\right]}^{-}$ is defined as ${\left[a\right]}^{-}=\max\left\{0,-a\right\}$. Connection weight $w_{kj}$ is defined as

(6) $$w_{kj}=f\left(d_{kj}\right)=\left\{\begin{array}{c} \frac{\mu }{d_{kj}},\;\;0<d_{kj}\leq r_{0},\\ 0,\;\;d_{kj}>r_{0}, \end{array}\right.$$

where μ is a positive constant and $d_{kl}$ represents the Euclidean distance between the *k*th neuron and the *l*th neuron. The neural activity is bounded in the area of [−D,B] as shown in Figure 1b. The parameters of the shunting model have been discussed in previous work [37]. The shunting model exhibits low sensitivity to both parameters and the neural connection weight function, which allows for a broad selection range of parameters. For the pursuit–evasion game, only two parameters *A* and μ are important factors. The neural network is very easy to saturate when parameter μ > 1. Thus, the value of μ is normally selected in the interval of μ∈(0,1]. When choosing a small *A* value, the small transient response makes the past influence of external inputs (pursuer or obstacle) disappear slowly. When choosing a big *A* value, the propagation from the current pursuer position becomes the domain contribution to the neuron activities.

**Theorem** **1.** *The steady-state neural activity is bounded in the area of [−D,B].*

**Proof** **of Theorem 1.** The shunting Equation (4) can be rewritten as

(7) $$\frac{dx_{k}}{dt}=\left(-A-S_{k}^{e}-S_{k}^{i}\right)x_{k}+BS_{k}^{e}-DS_{k}^{i},$$

where $dx_{k}/dt$ is linearly related to $x_{k}$ and this relationship can be categorized into three distinct scenarios. Firstly, when considering the absence of any inputs ($S_{k}^{i}=S_{k}^{e}=0$), the relationship can be described as

(8) $$\frac{dx_{k}}{dt}=-Ax_{k}.$$

When $x_{k}>0$, $dx_{k}/dt$ is negative and its magnitude increases as $x_{k}$ increases. In contrast, when $x_{k}<0$, $dx_{k}/dt$ is positive and its magnitude increases with decreasing $x_{k}$. As a result, at steady state ($dx_{k}/dt=0$), the value of $x_{k}$ converges to 0. Secondly, when only the presence of excitatory input is considered ($S_{k}^{i}=0$ and $S_{k}^{e}\neq 0$), the relationship can be described as

(9) $$\frac{dx_{k}}{dt}=\left(-A-S_{k}^{e}\right)x_{k}+BS_{k}^{e};$$

when $x_{k}$ is at the steady state ($dx_{k}/dt=0$), the value of $x_{k}$ can be given as

(10) $$x_{k}=B\frac{S_{k}^{e}}{A+S_{k}^{e}},$$

where *A* is a positive constant. Then, $A+S_{k}^{e}>S_{k}^{e}$; thus, $x_{k}$ converges to $BS_{k}^{e}/\left(A+S_{k}^{e}\right)<B$. Finally, when only the presence of inhibitory input is considered ($S_{k}^{i}\neq 0$ and $S_{k}^{e}=0$), the relationship can be described as

(11) $$\frac{dx_{k}}{dt}=\left(-A-S_{k}^{i}\right)x_{k}-DS_{k}^{i};$$

when $x_{k}$ is at the steady state ($dx_{k}/dt=0$), the value of $x_{k}$ can be given as

(12) $$x_{k}=-D\frac{S_{k}^{i}}{A+S_{k}^{i}},$$

where $A+S_{k}^{i}>S_{k}^{i}$; thus, $x_{k}$ converges to $-DS_{k}^{i}/(A+S_{k}^{i})>-D$. Therefore, neural activity $x_{k}$ is bounded within an interval of [−D,B]. □

### 3.2. Evasion Strategy Based on Neurodynamics

The directions and strategies of the pursuer and the evader are unknown to each other. Therefore, the evasion strategy cannot be guaranteed to be optimal because the strategy considers only the current locations of the players and not their locations at future times. The external input $I_{k}$ to the *k*th neuron is defined as

(13) $$I_{k}=\left\{\begin{array}{cc} E, & \mathrm{if}\;\mathrm{it}\;\mathrm{is}\;a\;\mathrm{pursuer},\\ -E, & \mathrm{if}\;\mathrm{it}\;\mathrm{is}\;\mathrm{an}\;\mathrm{obstacle},\\ 0, & \mathrm{otherwise}, \end{array}\right.$$

where *E* is a positive constant. If the corresponding position of the neuron is the pursuer, the external input becomes a large positive value. If the corresponding position is the obstacle, the external input becomes a large negative value. As the directions and strategies of the pursuer and evader are not mutually known, each evader assumes that the pursuer is pursuing itself specifically. The evasion strategy for each evader can be given as

(14) $$P_{e}⇐x_{P_{e}}=\min\left\{x_{j},l=1,2,\ldots ,n;\;x_{j}\geq 0\right\},$$

where $P_{e}$ represents the next position of the evader robot and $x_{P_{e}}$ represents the neural activity of the command neuron. From (5), there are two components in the excitatory term $S_{k}^{e}$. The ${\left[I_{k}\right]}^{+}$ term depends on the corresponding position of the pursuer, and the $\sum_{j=1}^{n}w_{kj}{\left[x_{j}\right]}^{+}$ term enables the propagation of positive activity to the whole neural network. The inhibitory term $S_{k}^{i}$ only consists of ${\left[I_{k}\right]}^{-}$, which depends only on the corresponding position of the obstacle. Therefore, the pursuer has global effects throughout the neural network, while the obstacle effect is local without propagation. As shown in Figure 1b, the neuron with the pursuer position has maximum neural activity and propagates the positive neural activity to its neighborhoods. The neurons of obstacles have negative neural activity without propagating.

**Theorem** **2.** *The trajectory of the evader is collision-free with the obstacles.*

**Proof** **of Theorem 2.** The neural activity of obstacle $x_{obs}$ at the steady state can be written as

(15) $$\begin{array}{c} x_{obs}=-D\frac{S_{k}^{i}}{A+S_{k}^{i}}<0. \end{array}$$

Since the evader only chooses the positive neural activity for the next position according to the evasion strategy (14), the obstacle neuron will not be chosen as the next position. □

**Theorem** **3.** *The proposed neural network has a real-time response to the obstacle.*

**Proof** **of Theorem 3.** Suppose that the *k*th neuron is not an obstacle at time instant $t_{1}$. Assume that one obstacle moves to the *k*th neuron at the instant of time $t_{2}$. The neural activity of the *k*th neuron at time instant $t_{2}$ can be written as

(16) $$\begin{array}{c} x_{k}\left(t_{2}\right)=x_{k}\left(t_{1}\right)+\frac{dx_{k}}{dt_{2}}=B\frac{\sum_{j=1}^{n}{\left[x_{j}\right]}^{+}}{d_{kj}}-DE\approx -E. \end{array}$$

Thus, for any time instant *t*, if the position of the neuron becomes the obstacle, the neural activity of this neuron changes to a very large negative value. Based on the evasion strategy (14), the evader would not choose the neuron with negative neural activity. Therefore, the trajectories of the pursuer and evader are collision-free in real time to the obstacle in any time instant. □

## 4. Results

To evaluate the performance of the proposed approach, simulations are performed with three types of obstacles. The positions of the pursuer, obstacles, and robots are randomly distributed in the workspace. The simulation studies are tested in MATLAB 2021a. The simulation parameters are listed as A=15, B=1, D=1, μ=1, E=50, $r_{0}=2$, $\lambda_{e}=3$, and $d_{c}=0.5$. The speed of obstacle $v_{o}$ is equal to that of the evader robot. The workspace is represented by a neural network that has 40×40 neurons.

### 4.1. Evasion with Static Obstacles

**Figure 2:**
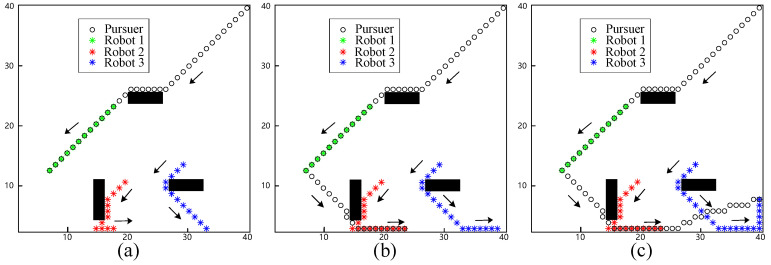
The evasion process with static obstacles. (**a**) The pursuer captures Robot 1 at the 12th step; (**b**) the pursuer captures Robot 2 at the 18th step; (**c**) the pursuer captures Robot 3 at the 24th step.

In the initial simulation, the proposed approach to avoid static obstacles was tested using one pursuer and three evader robots. The initial positions of the robots and the pursuer were as follows: the robots are located at (20, 23), (16, 10), and (30, 14), and the pursuer is located at (40, 40). The pursuer captured the closest robot, Robot 1, at the 12th step, as shown in Figure 2a. Subsequently, the pursuer captured Robot 2 at the 18th step, as shown in Figure 2b. At the beginning of the evasion, Robot 2 kept the same direction as Robot 1 but was later impeded by an obstacle. Lastly, Robot 3 was captured by the pursuer at the 24th step, as shown in Figure 2c. During the evasion process, both Robots 2 and 3 altered their evasion direction at the 9th and 15th steps, respectively. When close to the boundary of the workspace, the robots changed direction to avoid collision.

### 4.2. Evasion with Moving Obstacles

**Figure 3:**
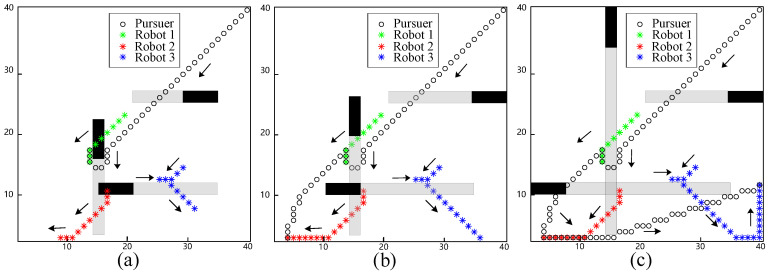
The evasion process with moving obstacles. (**a**) The pursuer captures Robot 1 at the 11th step; (**b**) the pursuer captures Robot 2 at the 16th step; (**c**) the pursuer captures Robot 3 at the 29th step.

In the next simulation, a more complex scenario was considered in which obstacles were moving within the workspace. In the simulation scenario, the velocity of the obstacles is equivalent to the evader robots. The gray lines represent the past trajectory of the obstacles. As shown in Figure 3a, Robot 1 was captured at the 11th step. An obstacle moving upward hindered the pursuit of Robot 1, leading it to move upward to evade the pursuer. Robot 2 was captured at the 16th step, as shown in Figure 3b. In contrast to the previous simulation, the obstacle started moving upward, providing enough space for Robot 2 to move toward the past position of the obstacle. Finally, Robot 3 was captured at the 29th step, as shown in Figure 3c. Initially, Robot 3 moved to the left as the obstacle blocked its evasion direction. However, as the pursuer closed in on Robot 1, Robot 3 reversed its direction and moved toward the right.

### 4.3. Evasion with Sudden Change Obstacles

**Figure 4:**
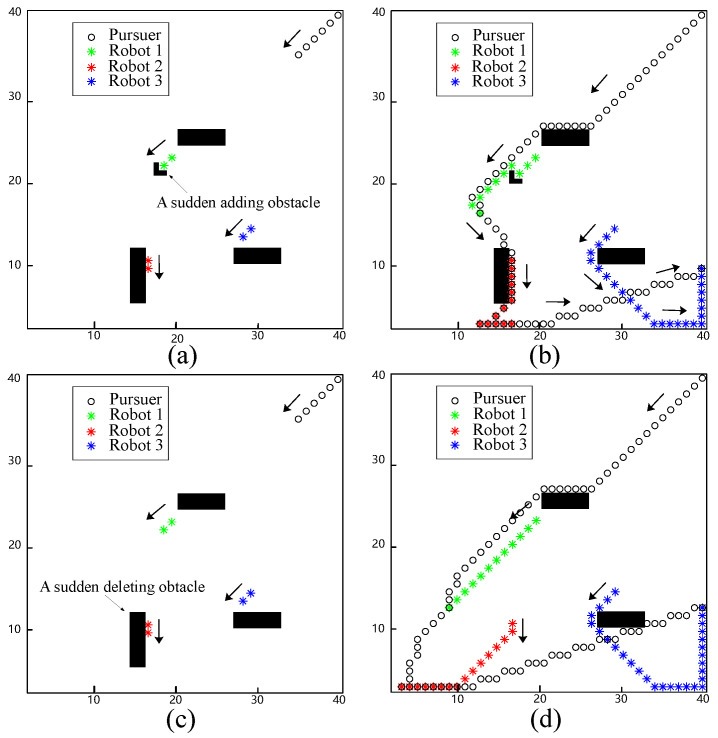
The evasion process with sudden change obstacles. (**a**) An L-shaped obstacle that suddenly adds to Robot 1; (**b**) the final evasion process with a suddenly adding obstacle; (**c**) a suddenly deleting obstacle near Robot 2; (**d**) the final evasion process with a suddenly deleting obstacle.

In this simulation, a case of sudden addition and removal of obstacles is tested to indicate the real-time response of the proposed approach. Figure 4a shows an L-shaped obstacle suddenly placed in front of Robot 1. Robot 1 is not able to move forward due to a sudden obstacle. The neural activity of the L-shaped obstacle immediately becomes a large negative value. Thus, Robot 1 moves to its right side and passes around obstacles, as shown in Figure 4b. Compared to the results shown in Figure 3b and Figure 4b, it can be observed that Robot 2 chose a different direction for evasion. This is because the sudden addition of the obstacle hindered the evasion of Robot 1, resulting in its early capture. As a result, the relative position of Robot 2 and the pursuer was greater than in the previous simulation, leading Robot 2 to move to the left to evade rather than to the right. Another scenario considered was the sudden removal of an obstacle. As shown in Figure 4c, an obstacle near Robot 2 was removed. After that, Robot 2 moved to the right, which increased the distance between the pursuers, as shown in Figure 4d.

### 4.4. Comparison Studies

In comparison studies, several experiments are tested in various scenarios to evaluate the performance of the proposed approach. The proposed approach and the compared approach are tested 30 times for different scenarios when $\lambda_{e}=3$. As mentioned in the literature review, the following approaches are compared with the proposed method:

1. CM: a collective moving approach is to form multiple evaders as a group moves together with others following a single leader. In this comparison study, a self-adaptive collective moving approach is used to guide evaders to move in the opposite direction of the pursuer [38].
2. LoS-PF: a potential field-based approach that generates attractive and repulsive forces to guide the evader [25]. The attractive force is based on the virtual target which is the line-of-sight direction of the pursuer, whereas the repulsive force is based on the position of obstacles.
3. RL: a reinforcement learning algorithm based on a self-play mechanism to guide the evader to escape from the pursuer and avoid collisions with obstacles [28]. The reward function of the evader is defined as follows:
  (17) $$R_{eva}=\left\{\begin{array}{cc} -10, & \mathrm{if}\;d_{eu}^{t}<d_{c},\\ -10, & \mathrm{if}\;\mathrm{collide}\;\mathrm{with}\;\mathrm{an}\;\mathrm{obstacle},\\ c(d_{eu}^{t}-d_{eu}^{t-1}), & \mathrm{otherwise}, \end{array}\right.$$
  where *c* is a small constant; $R_{eva}$ is the reward value of the evader in self-play training, and $d_{eu}^{t}$ is the distance between the pursuer and the evader at time *t*.

As shown in Table 1, the comparison results indicate that the proposed method obtains a longer survival time in dynamic scenarios, including moving, adding, and deleting environments, while the RL method outperforms other methods only in static scenarios. It is important to note the limitation of the RL method in terms of training time. Due to the position changes of the pursuer and the obstacle, the training process is restarted at every step. Therefore, the training time is significantly extended with an increasing number of evaders or environmental changes, which might pose significant challenges in real-world applications, where computational resources, time constraints, and the need for rapid deployment can limit the feasibility of the methods. However, the training process is not necessary in the proposed approach. The next evasive movement is based on the dynamic change in neural activity. It is unnecessary to incorporate a learning mechanism to determine optimal neural weights, which would lead to greater complexity of the algorithm and increased computational costs. Compared to the LoS-PF method, the evasion of the proposed method is based on the propagation of neural activity, which is not limited to a specific evasive direction. In contrast, the LoS-PF method is a direction-selective evasion strategy, where the chosen direction is the opposite of the pursuer. The opposite direction of the pursuer is the optimal direction for the evader in a collision-free environment. However, in environments with obstacles, a singular escape direction may lead the evader to a local minimum constituted by obstacles, which leads the evader to be trapped at the current location. This limitation is also present in the CM approach that involves multiple evaders moving as a group with a single leader, which can lead to a group of evaders in a single direction.

### 4.5. Real-World Experiments on Mobile Robots

**Figure 5:**
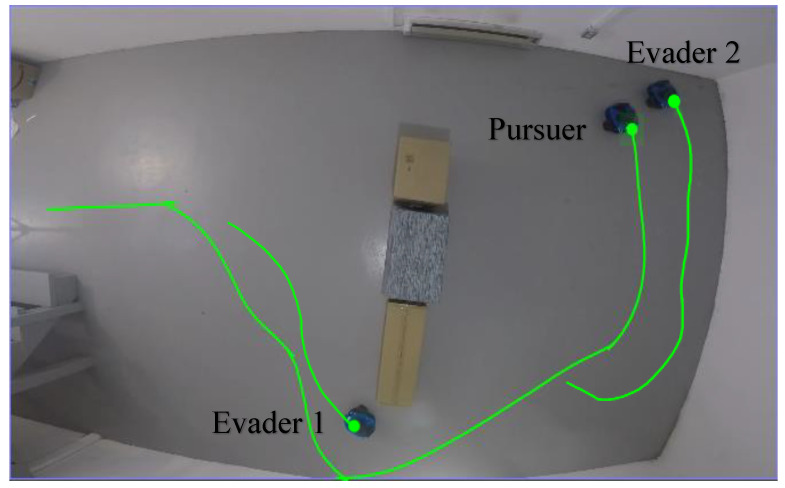
The real-world experiment for validating the performance of the proposed method using three mobile robots.

To validate the proposed neurodynamic-based evasion method for mobile robots, three robot operating systems (ROSs) were built for experimental tests, as shown in Figure 5. Each robot was equipped with a 1080p camera and an RPLIDAR A1 laser scanner and both were integrated into an Ubuntu system. For control, environment detection, and localization tasks, the robots used Raspberry Pi 4 Model B and STM32F405 computing boards. The proposed evasion strategy can be considered as a virtual target path planning for robots. When the pursuit–evasion game begins, the command position of the evader $P_{e}=\left(x_{e},y_{e}\right)$ is sent to mobile robots according to the environment and the current position of the pursuer. Evader robots set the command position as the current virtual target for path planning. The experimental results are in reasonable agreement with the simulation results.

## 5. Conclusions

This paper presents a novel neurodynamics-based approach that addresses the general pursuit–evasion game from a neurodynamic perspective instead of formulating it as a differential game. A specific evasion task considered the case in which one single pursuer moves faster than the evader robots. The approach utilizes a BINN with only local connections, which is topologically organized to represent the environment. The pursuer has global effects on the whole neural network, whereas the obstacles only have local effects to guarantee evader robots avoid collisions. The proposed approach is capable of generating collision-free evasion trajectories and has a real-time response to the changing environment through dynamic neural activity. The simulation and experimental results demonstrate that the proposed approach is effective and efficient in complex and dynamic environments. The limitation of the proposed method can be summarized as follows. Firstly, the evasion direction in the proposed method is constrained to eight fixed directions, whereas the evasive direction of the real robot can be finely adjusted. Secondly, the proposed method assumes that the robots are point robots, which ignores the dynamics of actual robotic systems. This limits the applicability and effectiveness of the method in practical settings. Future work will aim to incorporate continuous adjustment of the direction of evasion and integrate realistic robotic models to improve applicability and effectiveness in real-world robotic evasion scenarios.

## Figures and Tables

**Table 1:** Comparison of the average survival time in different scenarios.

Approach	Static	Moving	Adding	Deleting
CM [38]	14.57	15.07	14.57	14.43
LoS-PF [25]	19.40	15.73	19.87	21.40
RL [28]	23.10	23.57	23.30	22.27
Proposed	21.97	24.70	23.77	23.80

The maximum survival time is highlighted in bold.

## Data Availability

Data are contained within the article.
